# Phosphorene/rhenium disulfide heterojunction-based negative differential resistance device for multi-valued logic

**DOI:** 10.1038/ncomms13413

**Published:** 2016-11-07

**Authors:** Jaewoo Shim, Seyong Oh, Dong-Ho Kang, Seo-Hyeon Jo, Muhammad Hasnain Ali, Woo-Young Choi, Keun Heo, Jaeho Jeon, Sungjoo Lee, Minwoo Kim, Young Jae Song, Jin-Hong Park

**Affiliations:** 1 School of Electronic and Electrical Engineering, Sungkyunkwan University, Suwon 440-746, Korea; 2 Frontier Technology Lab, R&D Headquarters, SK Hynix Co. Ltd., Ichon 460-701, Korea; 3 Sungkyunkwan University Advanced Institute of Nanotechnology, Sungkyunkwan University, Suwon 440-746, Korea

## Abstract

Recently, negative differential resistance devices have attracted considerable attention due to their folded current–voltage characteristic, which presents multiple threshold voltage values. Because of this remarkable property, studies associated with the negative differential resistance devices have been explored for realizing multi-valued logic applications. Here we demonstrate a negative differential resistance device based on a phosphorene/rhenium disulfide (BP/ReS_2_) heterojunction that is formed by type-III broken-gap band alignment, showing high peak-to-valley current ratio values of 4.2 and 6.9 at room temperature and 180 K, respectively. Also, the carrier transport mechanism of the BP/ReS_2_ negative differential resistance device is investigated in detail by analysing the tunnelling and diffusion currents at various temperatures with the proposed analytic negative differential resistance device model. Finally, we demonstrate a ternary inverter as a multi-valued logic application. This study of a two-dimensional material heterojunction is a step forward toward future multi-valued logic device research.

## Abstract (web summary)

Electronic devices based on negative differential resistance hold promise for multi-valued logic applications. Here, the authors implement such functionalities using an atomically thin phosphorene/rhenium disulfide van der Waals heterostructure, and further demonstrate the implementation of a ternary inverter.

## Graphical abstract

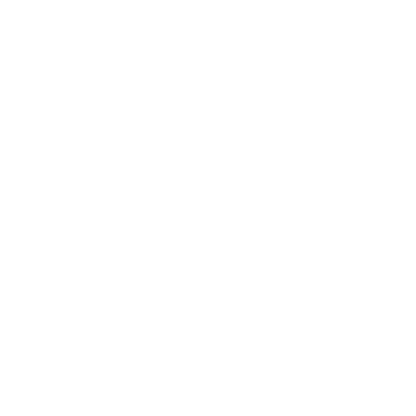

Recently, negative differential resistance (NDR) devices have attracted considerable attention owing to their folded current–voltage (*I*–*V*) characteristic (N-shaped *I*–*V* curve), which presents multiple threshold voltage values^1^,^2^,^3^,^4^,^5^,^6^,^7^,^8^,^9^,^10^,^11^,^12^,^13^,^14^,^15^,^16^,^17^,^18^,^19^,^20^,^21^,^22^,^23^,^24^,^25^,^26^,^27^. Because of this remarkable property, studies associated with the NDR devices have been explored for realizing multi-valued logic (MVL) applications^1^,^7^,^11^,^13^,^26^. Compared to conventional binary logic systems, MVL systems can transmit more information with fewer interconnect lines between devices by transferring multi-valued signals, thereby reducing the complexity of modern integrated circuit design. For example, a ternary logic system is theoretically able to reduce the number of interconnect lines by nearly 45% as compared with binary logic^28^. The NDR devices that have been researched for the implementation of this MVL system are Esaki diodes^2^,^3^,^4^,^5^,^6^,^7^, resonant tunnelling diodes^8^,^9^,^10^,^11^,^12^,^13^,^14^,^15^,^16^,^17^,^18^,^19^,^20^, Gunn diodes, single electron transistors^21^,^22^ and molecular devices^23^,^24^. However, at the current stage of research, because most of the Esaki diodes and resonant tunnelling diodes were fabricated in Si–Ge and III–V semiconductors^2^,^3^,^4^,^8^,^9^,^10^,^11^,^12^,^13^,^14^, the formation of various types of heterojunctions (type-I, II and III) is limited by threading dislocations, which are normally caused at the junction interface by lattice mismatch during film growth. Although the threading dislocation that increases the valley current of NDR device can be reduced by applying superlattice and nanowire structures, it is hard to avoid that the fabrication process becomes more complex.

In this light, atomically thin two-dimensional (2D) semiconductors, such as molybdenum disulfide (MoS_2_), tungsten diselenide (WSe_2_), rhenium disulfide (ReS_2_), tin diselenide (SnSe_2_) and black phosphorus (BP), are expected to offer attractive material platforms for NDR devices due to the absence of dangling bonds on their surfaces. Because these 2D semiconductor layers are stacked via weak van der Waals interaction, 2D materials-based heterojunctions do not suffer from lattice mismatch and form atomically sharp interfaces, allowing high-quality heterojunction interfaces^29^,^30^,^31^. It is also possible to design various heterojunctions by stacking different 2D materials with different bandgaps and electron affinities, where band structure alignment can be classified into three types: type-I (straddling gap)^32^, type-II (staggered gap)^2^,^3^,^4^,^6^,^7^,^33^,^34^,^35^,^36^ and type-III (broken gap)^5^,^32^. Recently, Roy *et al*.^6^ reported an NDR device based on an MoS_2_/WSe_2_ heterostructure, which was fabricated in a type-II heterojunction. However, the NDR device used dual gates involving a complicated fabrication process to obtain an electrostatically doped n^+^/p^+^ heterojunction, and the NDR behaviour was observed at a very low temperature below 175 K. Nourbakhsh *et al*. and Yan *et al*. also reported NDR devices in MoS_2_/WSe_2_ and BP/SnSe_2_ heterojunctions, respectively^5^,^7^. In these devices, it was necessary to use the specific thickness of 2D semiconductors to ensure band-to-band tunnelling of carriers, and the obtained peak-to-valley current ratio (PVCR) values were lower than 2 at room temperature.

Here, we demonstrate an NDR device based on a BP/ReS_2_ heterojunction that is formed by type-III broken-gap band alignment, showing high PVCR values of 4.2 and 6.9 at room temperature and 180 K, respectively. In addition, as an MVL application, we present a ternary inverter (having three states) that combines a BP/ReS_2_ heterojunction NDR device and a BP p-channel thin film transistor (TFT). This integration approach based on NDR devices is expected to fulfil low-power advantages of future MVL circuits by reducing the parasitic interconnect capacitance. In particular, compared with a type-II heterojunction, a type-III heterojunction can easily implement a highly doped n^+^/p^+^ heterojunction without a separate process, such as electrostatic doping by gate bias and a chemical doping process. First, we confirm the broken-gap band alignment of the BP/ReS_2_ heterojunction with Kelvin probe force microscopy (KPFM). Then, the carrier transport mechanism of the BP/ReS_2_ heterojunction NDR device is discussed in detail at room temperature. Furthermore, through temperature-dependent current–voltage (*I*–*V*) measurements and the proposed analytic NDR device model, where tunnelling/diffusion currents and parasitic series resistance were considered simultaneously, we quantitatively study the temperature-dependent device operations.

## Results

### Characteristics of BP/ReS_2_ heterostructure

**Figure 1:**
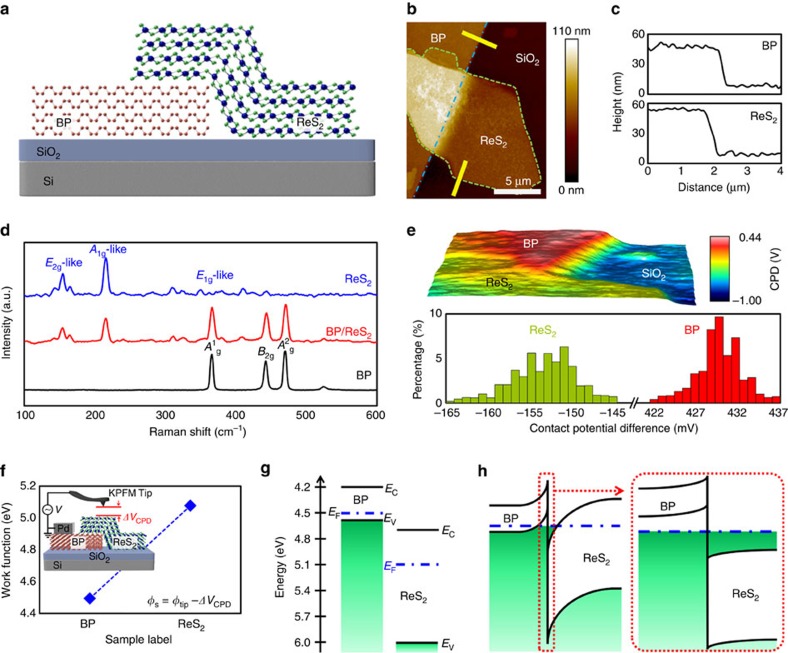
BP/ReS_2_ heterostructure. (**a**) Schematic illustration of the BP/ReS_2_ heterostructure on SiO_2_/Si substrate. (**b**) AFM (atomic force microscope) image of the BP/ReS_2_ heterostructure sample. (**c**) Thicknesses of the BP (top) and the ReS_2_ flakes (bottom) corresponding to the yellow lines marked in **b**. (**d**) Raman spectra of the ReS_2_, BP/ReS_2_ overlapped and BP regions. (**e**) Three-dimensional KPFM mapping image of the BP/ReS_2_ heterostructure (top) and histogram distributions of Δ*V*_CPD_ extracted from the KPFM mapping image (bottom). (**f**) Work function values of BP and ReS_2_ films. The inset shows schematic illustration of the KPFM measurement system. (**g**,**h**) Energy band alignments of BP and ReS_2_ heterojunction at equilibrium (**g**) before and (**h**) after contact. *E*_C_, *E*_F_ and *E*_V_ are the lowest energy level of the conduction band, the Fermi level and the highest energy level of the valence band of the semiconductors, respectively.

Figure 1a presents schematic diagrams of the BP/ReS_2_ heterostructure on an SiO_2_/Si substrate. A BP flake was prepared onto an SiO_2_/Si substrate by a tape-based exfoliation method^37^, and then an ReS_2_ flake was transferred onto the BP flake by a mechanical transfer process method (the optical images of the BP/ReS_2_ heterostructure can be found in Supplementary Fig. 1)^38^. The thicknesses of the BP and ReS_2_ flakes were confirmed by atomic force microscope to be about 40 and 50 nm, respectively (Fig. 1b,c). Figure 1d shows the Raman spectra obtained at three different positions in the BP/ReS_2_ heterostructure sample, where the spectra from top to bottom indicate a ReS_2_ region, a BP/ReS_2_ overlapped region and a BP region. The observed Raman peaks of BP at 366, 442 and 470 cm^−1^ correspond to the *A*^1^_g_, *B*_2g_, and *A*^2^_g_ phonon modes, respectively. This Raman spectrum for ReS_2_ includes two prominent peaks at 154 and 215 cm^−1^, which are attributed to the in-plane (*E*_2g_) and out-of-plane (*A*_1g_) vibrational modes. The Raman spectrum of the overlapped BP/ReS_2_ region contains the vibration modes of both BP and ReS_2_, indicating the formation of a heterostructure. Next, to investigate the band alignment of the BP/ReS_2_ heterojunction, we carried out KPFM measurements. Figure 1e shows the three-dimensional KPFM mapping image of the BP/ReS_2_ heterostructure and contact potential difference (Δ*V*_CPD_) histograms extracted from the mapping image. Before the KPFM measurement, the KPFM tip (platinum/iridium (Pt/Ir)-coated Si tip) was calibrated on a highly oriented pyrolytic graphite (HOPG) surface. Here, the HOPG is conventionally used to calibrate the work function of the KPFM tip because it has a clean surface and its work function is well known to be 4.6 eV (ref. 39). The average Δ*V*_CPD_ values on the BP and ReS_2_ flakes were obtained at −153 and 430 mV, respectively. Since the Δ*V*_CPD_ is the difference in the work function between the KPFM tip and the sample (inset of Fig. 1f), the work function values of the BP and ReS_2_ can be calculated using the following equation: *Φ*_s_=*Φ*_tip_−Δ*V*_CPD_, where *Φ*_s_ and *Φ*_tip_ are the work functions of the samples (BP and ReS_2_) and the KPFM tip, respectively. Here, *Φ*_tip_ was obtained from the sum of the HOPG work function (*Φ*_HOPG_) and Δ*V*_CPD_ between the KPFM tip and the HOPG surface (*Φ*_tip_=*Φ*_HOPG_+Δ*V*_CPD_HOPG_), which is presented in more detail in Supplementary Fig. 2 (refs 40, 41). Therefore, the work function values of the BP and ReS_2_ films can be estimated to be about 4.5 and 5.1 eV, respectively (Fig. 1f). Based on the obtained KPFM results and the previously reported band properties (conduction band minimum, valence band maximum and band gap (*E*_g_)) of BP and ReS_2_ (refs 42, 43, 44), we graphically described the predicted energy band alignment of the BP and ReS_2_ heterojunction at equilibrium before contact (Fig. 1g) and after contact (Fig. 1h). Here, the conduction band minimum, valence band maximum and *E*_g_ values of the BP (ReS_2_) that were calculated using a first-principles density of states in the literature were 4.2 eV (4.68 eV), 4.59 eV (6.05 eV) and 0.39 eV (1.37 eV), respectively. As shown in Fig. 1g, the BP/ReS_2_ heterojunction seems to form a broken-gap band alignment (type-III heterojunction) because the highest valence band edge of BP is located above the lowest conduction band edge of ReS_2_. Furthermore, owing to the large work function difference (0.6 eV) between BP and ReS_2_, hole and electron carriers accumulate near the heterojunction interface in BP and ReS_2_, respectively (Fig. 1h). Therefore, a highly doped n^+^/p^+^ heterojunction can easily be implemented by forming a broken-gap band alignment without using a separate doping process, such as electrostatic doping by gate bias or chemical doping, which is generally required in a type-II heterojunction to realize a NDR device^2^,^3^,^4^,^6^,^7^,^33^,^34^,^35^,^36^.

### BP/ReS_2_ heterojunction-based NDR device

**Figure 2:**
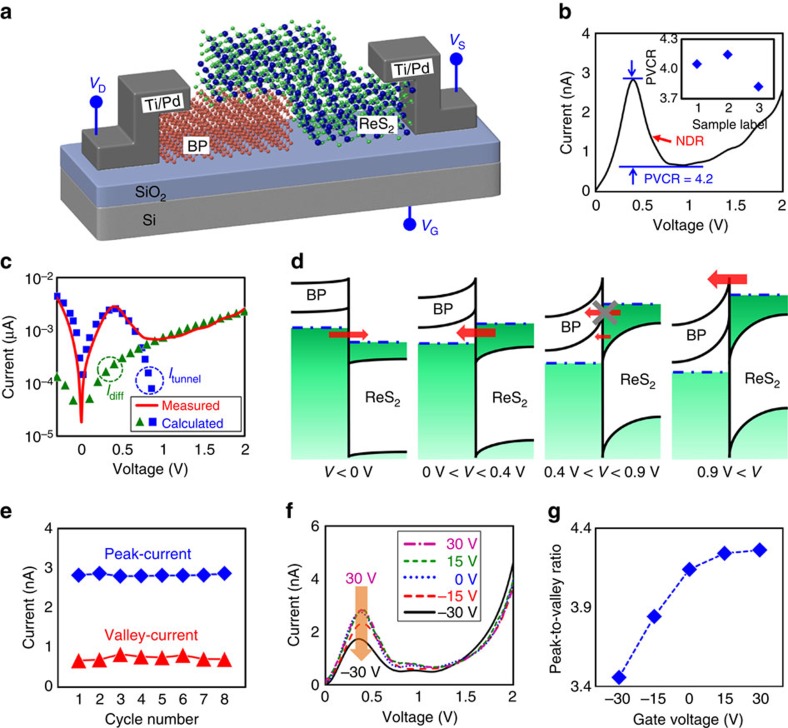
Electrical characteristics of BP/ReS_2_ heterojunction-based NDR device at room temperature. (**a**) An illustration of the BP/ReS_2_ heterojunction NDR device. (**b**) Current–voltage (*I*–*V*) characteristic of the BP/ReS_2_ NDR device on a linear scale. The inset shows the PVCR values for the three different BP/ReS_2_ NDR devices. (**c**) Experimentally measured and theoretically calculated *I*–*V* curves of the BP/ReS_2_ NDR device on a log scale. (**d**) Energy band alignment of the BP/ReS_2_ heterojunction under various bias conditions. Width of the red arrow presents the magnitude of the current. (**e**) Extracted peak- and valley-current values of the BP/ReS_2_ NDR device in eight consecutive *I*–*V* sweeps. (**f**) Drain current–drain voltage (*I*_D_–*V*_D_) curves under various gate biases from 30 V to −30 V. (**g**) PVCR values of the BP/ReS_2_ NDR device as a function of gate voltage.

After fabricating the NDR device based on the BP/ReS_2_ heterojunction, as shown in Fig. 2a, we performed electrical measurements in the NDR device at room temperature. Figure 2b shows the current–voltage (*I*–*V*) characteristic of the NDR device on a linear scale. Here, the NDR behaviour was observed between 0.4 V and 0.9 V with a PVCR of 4.2, which is the highest value in previously reported NDR devices based on 2D materials^5^,^6^,^7^,^16^,^17^,^18^,^19^. We also note that similar electrical characteristics were observed in three different BP/ReS_2_ NDR devices with PVCR values between 3.8 and 4.1 (Inset of Fig. 2 and Supplementary Fig. 3). In addition, to understand the operation mechanism of the BP/ReS_2_ NDR device, we theoretically investigated the current characteristic by considering tunnelling and diffusion currents using a theoretical model that we developed. The equations related to the current transport mechanisms can be found in Supplementary Fig. 4 and the parameters used in the analytic model are tabulated in Supplementary Table 1. The experimentally measured and theoretically calculated *I*–*V* curves are shown in Fig. 2c. Under a negative voltage and a positive voltage between 0 and 0.7 V, the tunnelling current seems to dominate the diffusion current, whereas the diffusion current primarily contributes to the operation of the NDR device when a higher voltage is applied (above 0.7 V). This is graphically explained in Fig. 2d, which shows the band alignments of the BP/ReS_2_ heterojunction under various bias conditions. When a negative voltage is applied (*V*<0 V), electron carriers are able to tunnel from the filled valence band states in BP to the empty conduction band states in ReS_2_, consequently increasing the current. Similarly, when a small positive voltage is applied (0 V<*V*<0.4 V), the current increases because the electron carriers in the conduction band states of ReS_2_ are tunnelled into the empty valence band states of BP. This current of the NDR device continuously increases until the Fermi level of ReS_2_ aligns with the highest valence band energy of BP, where the filled states in the ReS_2_ are maximally overlapped with unoccupied states of the BP, inducing a maximum tunnelling current (peak current). Further increases in voltage (0.4 V<*V*<0.9 V) lead to decreases in the current because the degree of overlap between the filled and empty states is reduced due to the bandgap region. Therefore, the tunnelling current decreases with increasing voltage, and the NDR behaviour is obtained as shown in Fig. 2b,c. When a high voltage is applied (*V*>0.9 V), the tunnelling current no longer affects the operation of the NDR device, and the electron carriers are able to diffuse from ReS_2_ to BP by shrinking the potential hill in the BP/ReS_2_ heterojunction, consequently again increasing the current of the BP/ReS_2_ NDR device. Here, the lowest current value that is observed beyond a peak current is called a valley current. We then extracted the peak- and valley-current values of the NDR device for eight consecutive *I*–*V* sweeps, where stable peak- and valley-current values were observed, as shown in Fig. 2e. Figure 2f shows the drain current–drain voltage (*I*_D_–*V*_D_) curves under various gate bias conditions, which also confirms that the peak current decreases as the gate voltage decreases. When the gate voltage varied from 30 V to −30 V, the Fermi level of BP down-shifted due to the accumulation of hole carriers, which thereby increased the degree of the energy band bending in the BP region (Supplementary Fig. 5). The Fermi level of the stacked ReS_2_ on the BP is predicted to be barely modulated by an applied gate bias due to the thick BP (strong electrostatic screening effect). The down-shifted energy band in the BP region would form a potential well at the heterojunction interface, where much higher potential barrier height was obtained^45^. This leads to a decrease in the peak current of the BP/ReS_2_ NDR device with decreasing gate voltage because strongly confined electron carriers in the potential well are difficult to escape from the potential well. The reduction of peak current in BP/ReS_2_ NDR devices with decreasing gate voltage could also be estimated using the *I*_D_–*V*_D_ curves calculated by the analytic model (Supplementary Fig. 5). Thus, the PVCR of the BP/ReS_2_ NDR device was modulated between 4.26 and 3.46A/A by applying different gate voltages, as shown in Fig. 2g.

**Figure 3:**
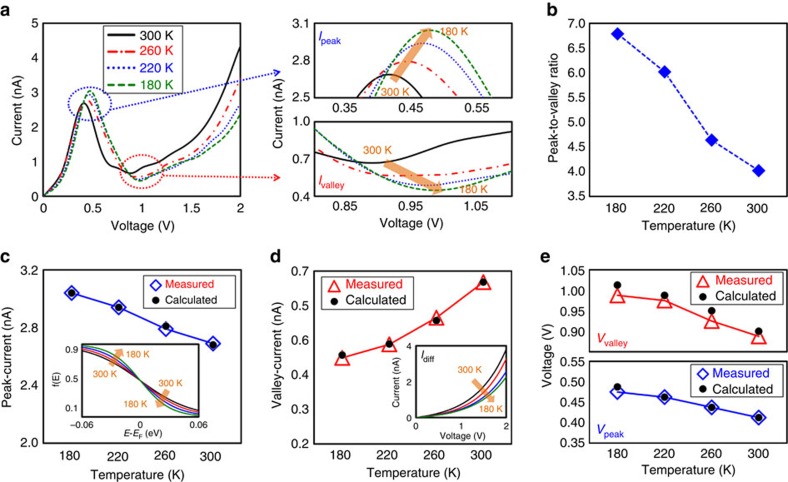
Temperature-dependent electrical characteristics of BP/ReS_2_ NDR device. (**a**) *I*–*V* curves of the BP/ReS_2_ NDR device at various temperatures between 180 K and 300 K. (**b**) PVCR values of the BP/ReS_2_ NDR device as a function of temperature. (**c**–**e**) Peak-current (**c**), valley-current (**d**), valley- and peak-voltage values of the BP/ReS_2_ NDR device as a function of temperature (**e**), which were extracted from the experimentally measured and the theoretically calculated *I*–*V* characteristic curves. The inset in **c** shows the probability of states being occupied (*f*(*E*)) as a function of given energy *E* relative to *E*_F_(*E*−*E*_F_). The inset in **d** shows the theoretically calculated diffusion current of the BP/ReS_2_ NDR device at various temperatures.

Furthermore, to analyse the temperature dependency of the carrier transport in the BP/ReS_2_ NDR device, we performed *I*–*V* measurements at various temperatures between 180 and 300 K. As shown in Fig. 3a, the peak current (*I*_peak_) increased, whereas the valley current (*I*_valley_) decreased, with reducing temperature, consequently improving PVCR value from 4.02 to 6.78A/A (Fig. 3b). In addition, the peak-voltage (*V*_peak_) and valley-voltage (*V*_valley_) values shifted positively as the measurement temperature was reduced. To quantitatively analyse the temperature-dependent electrical characteristics of the BP/ReS_2_ NDR device, we exploited the proposed analytic NDR device model. The calculated *I*–*V* characteristic curves at different temperatures are presented in Supplementary Fig. 6, where the *I*–*V* curves estimated by the analytic model were well fitted with the measured *I*–*V* data. Figure 3c shows the *I*_peak_ data as a function of temperature, which were extracted from the experimentally measured and the theoretically calculated *I*–*V* characteristics. Because a large portion of *I*_peak_ is mainly occupied by *I*_tunnel_, as shown in Fig. 2c, the *I*_peak_ seems to be associated with the density of states in the conduction band of ReS_2_ and the valence band of BP, where the density of occupied or empty states is determined by the Fermi–Dirac function. Thus, we focused on an analysis on the temperature dependency of the Fermi–Dirac distribution. As the temperature decreases, the Fermi–Dirac distribution near the Fermi level of BP and ReS_2_ becomes sharp, thereby increasing the probability of states being occupied (*f*(*E*)) in the conduction band of ReS_2_, as shown in the inset of Fig. 3c, where *f*(*E*) at energy *E* of *E*_F_−0.03 eV were 0.76 and 0.87 at 300 K and 180 K, respectively. Meanwhile, the *f*(*E*) in the valence band of BP decreases (thereby, an increase in probability of states being empty) with reducing the temperature, where *f*(*E*) at energy *E*=*E*_F_+0.03 eV were 0.24 and 0.13 at 300 and 180 K, respectively. This subsequently increases *I*_tunnel_ because of the increased occupied states in conduction band of ReS_2_ and the decreased empty states in valence band of BP, eventually resulting in a slight increase of *I*_peak_ (2.7 nA at 300 K and 3.0 nA at 180 K in Fig. 3c). In contrast, because the dominant current of *I*_valley_ is *I*_diff_, which is dependent on temperature (see the inset of Fig. 3d), *I*_valley_ is predicted to reduce with decreasing temperature (0.67 nA at 300 K and 0.45 nA at 180 K, in Fig. 3d). Overall, in the BP/ReS_2_ NDR device, the temperature dependencies of *I*_peak_ and *I*_valley_ were differently presented due to the increase in *I*_tunnel_ and the decrease in *I*_diff_ as the measurement temperature decreased. Meanwhile, we also considered parasitic series resistance (*R*_s_) in the analytic model to accurately analyse the device operation. *R*_s_ is mainly associated with the contact resistance between the metal electrode and the semiconductor^46^. Here, the reduction in n-type carrier concentration due to decreasing temperature leads to an increase in the depletion width at the metal/semiconductor (MS) junction and thereby a suppression of the e-field-dependent barrier height lowering effect, eventually increasing the contact resistance at the MS junction (Supplementary Fig. 7)^47^. Thus, as shown in Fig. 3e, positively shifted *V*_peak_ and *V*_valley_ were observed as measurement temperature decreased because a higher voltage was required to operate the NDR device due to the increased *R*_S_ at reduced temperature.

### Ternary inverter with three logical states

**Figure 4:**
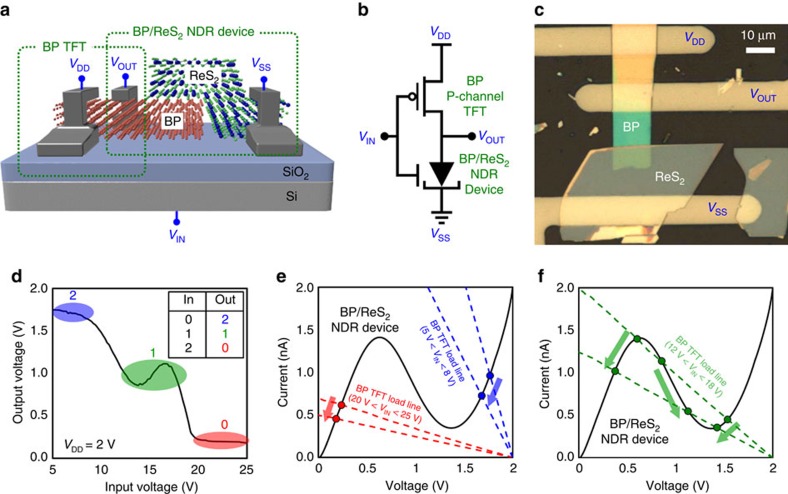
Ternary inverter with three logical states. (**a**) Schematic illustration of the ternary inverter. (**b**) Equivalent circuit configuration of the ternary inverter. (**c**) Optical image of the ternary inverter. (**d**) *V*_IN_ versus *V*_OUT_ characteristic of the ternary inverter. The inset shows an input–output table of the ternary inverter. (**e**,**f**) Load-line analysis of the ternary inverter circuit under three bias conditions: (**e**) 5 V<*V*_IN_<8 V, 20 V<*V*_IN_<25 V and (**f**) 12 V<*V*_IN_<18 V. The *I*–*V* characteristics of the BP/ReS_2_ NDR device (driver) and the BP TFT (load resistor) are represented by solid and dashed lines, respectively.

Finally, we fabricated a ternary inverter, which is a basic building block in MVL applications, as schematically shown in Fig. 4a. This ternary inverter was formed by integrating the BP/ReS_2_ heterojunction NDR device as a driver with the built-in BP p-channel TFT as a load resistor, where the total resistance in the BP TFT could be controlled by an applied gate voltage (Supplementary Fig. 8). Figure 4b,c show the equivalent circuit configuration and an optical image of the ternary inverter, respectively. The supply (*V*_DD_) and input voltages (*V*_IN_) were applied to the source electrode on the BP and the back gate. The metal electrode on the ReS_2_ (source electrode in the BP/ReS_2_ NDR device) was connected to the ground (*V*_SS_), and then we measured the output voltage (*V*_OUT_) on the middle shared electrode (drain electrode in the BP TFT and in the BP/ReS_2_ NDR device). The *V*_IN_ versus *V*_OUT_ characteristic of the ternary inverter is shown in Fig. 4d, where *V*_DD_ was 2 V. When *V*_IN_ varied from 5 V to 25 V, *V*_OUT_ showed three distinct states: (i) *V*_OUT_>1.7 V (state ‘2') for 5 V<*V*_IN_<8 V, (ii) 0.85 V<*V*_OUT_<1.12 V (state ‘1') for 12 V<*V*_IN_<18 V and (iii) *V*_OUT_<0.24 V (state ‘0') for 20 V<*V*_IN_<25 V. To explain the operation of this ternary inverter, we performed a load-line circuit analysis, in which the intersections of the two characteristic curves indicate the operating points of this circuit. As shown in Fig. 4e, when a low *V*_IN_ is applied (5 V<*V*_IN_<8 V), the load resistor (BP TFT) provides a low-resistance path between the source (*V*_DD_) and drain (output) nodes of the BP TFT because the applied *V*_IN_ is higher than the threshold voltage (*V*_TH_) of the BP TFT (Supplementary Fig. 8). Thus, high voltage values (logic state ‘2'), which were close to *V*_DD_, were measured at the output terminal (blue circles in Fig. 4e). In contrast, when a high *V*_IN_ was applied (20 V<*V*_IN_<25 V), the BP TFT was turned off (*V*_IN_<*V*_TH_), which creates a low-resistance path between the output terminal and the ground. This consequently presented low voltage values (logic state ‘0') at the output terminal (red circles in Fig. 4e). When a moderate *V*_IN_ was applied (12 V<*V*_IN_<18 V), the operating points were located at the NDR region in the *I*–*V* curve of the BP/ReS_2_ NDR device, as shown in Fig. 4f. This resulted in intermediate output values (logic state ‘1') with small fluctuations due to an imbalance of the operating points, where the three intersections were. Overall, by integrating the BP/ReS_2_ NDR device with the built-in BP TFT, the ternary inverter was simply demonstrated as an MVL application.

## Discussion

We demonstrated a NDR device based on a BP/ReS_2_ heterojunction with high PVCR values of 4.2 and 6.8 at room temperature and 180 K, respectively. This NDR characteristic can be easily achieved by forming a broken-gap (type-III) band alignment without a separate process, such as electrostatic doping by gate bias and a chemical doping process, which is generally required in type-II heterojunction to realize an NDR device. The broken-gap band alignment of the BP/ReS_2_ heterojunction was confirmed through KPFM measurements, where the band gaps of the BP and ReS_2_ did not overlap at all (type-III). Also, the carrier transport mechanisms of the BP/ReS_2_ NDR device were investigated in detail by analysing the tunnelling and diffusion currents at various temperatures between 180 and 300 K by using the proposed analytic NDR device model. Specifically, we confirmed that *I*_peak_ increased while *I*_valley_ decreased as the measurement temperature was reduced, consequently providing a PVCR value that improved from 4.02 to 6.8. Finally, we demonstrated a ternary inverter as an MVL application, which was fabricated by integrating a BP/ReS_2_ heterojunction NDR device with a built-in BP TFT. In the *V*_IN_ versus *V*_OUT_ characteristic of the ternary inverter, when *V*_IN_ varied from 5 to 25 V, *V*_OUT_ showed three distinct values (states ‘2', ‘1' and ‘0'). This study of a 2D material heterojunction is a step forward toward future multi-valued logic device research.

## Methods

### Fabrication of the BP/ReS_2_ heterojunction-based NDR devices

A BP flake was exfoliated onto a 90 nm thick SiO_2_/Si substrate by adhesive tape (224SPV, Nitto). Then, a ReS_2_ flake was transferred onto the BP flake by using a mechanical transfer process method. Finally, the source and drain electrode regions were patterned by optical lithography, and Ti/Pd (10/30 nm) layers were deposited on an electron-beam evaporating system, followed by a lift-off process.

### Fabrication of the ternary inverter

By using a mechanical transfer method, a ReS_2_ flake was stacked onto the BP flake, which was exfoliated onto a 90 nm thick SiO_2_/Si substrate. The metal electrode regions were defined using a conventional photolithography process. Finally, Ti/Pd (10/30 nm) layers were deposited by e-beam evaporation to form the contacts for BP and ReS_2_, followed by a lift-off process in acetone. The BP/ReS_2_ NDR and the BP TFT devices were designed to function as a driver and a load resistor for a ternary inverter, respectively. The voltage of *V*_DD_ was applied to the source electrode of the BP TFT, and the source electrode of the BP/ReS_2_ NDR device was connected to the ground (*V*_SS_). The common back gate of the BP TFT and BP/ReS_2_ NDR devices served as the input voltage (*V*_IN_) electrode. The output voltage (*V*_OUT_) was measured at the drain electrode of the BP/ReS_2_ NDR device.

### Characterization of the BP/ReS_2_ heterojunctions

Raman studies were conducted using a WITec micro-Raman spectrometer system with a frequency-doubled Nd-YAG laser beam (532 nm laser excitation). The atomic force microscope analysis was carried out in an XE 100 (Park Systems Corp.) system. The electrical transport measurements were conducted at room temperature under ambient conditions in a probe station with a Keysight B2912A. The temperature-dependent electrical characteristics were measured in a vacuum chamber (below 10^−4^ Torr) using a Keithley 4200 Semiconductor Parameter Analyzer. The KPFM measurement was performed using NTEGRA Spectra (NT-MDT).

### Theoretic model of carrier transport in BP/ReS_2_ heterojunctions

The tunnelling current (*I*_tunnel_) and diffusion current (*I*_diff_) were considered to understand the operating mechanism of the BP/ReS_2_ NDR device. The *I*_tunnel_ can be obtained from


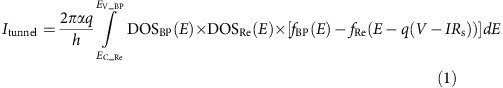


where *α* is the screening factor, *q* is the elementary charge, *h* is the Planck constant, *E*_V_BP_ is the highest valence band energy in BP, *E*_C_Re_ is the lowest conduction band energy in ReS_2_. DOS_BP_(*E*), DOS_Re_(*E*), *f*_BP_(*E*) and *f*_Re_(*E*) mean the density of states and Fermi–Dirac distribution functions of BP and ReS_2_, respectively.

The *I*_diff_ is obtained from


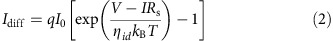


where *I*_0_ is the saturation current, *V* is the applied voltage, *I* is the junction current, *R*_s_ is the series resistance, *η*_id_ is the ideality factor, *k*_B_ is the Boltzmann constant and *T* is the temperature.

### Data availability

The data that support the findings of this study are available from the corresponding author upon request.

## Additional information

**How to cite this article:** Shim, J. *et al*. Phosphorene/rhenium disulfide heterojunction-based negative differential resistance device for multi-valued logic. *Nat. Commun.* **7,** 13413 doi: 10.1038/ncomms13413 (2016).

**Publisher's note:** Springer Nature remains neutral with regard to jurisdictional claims in published maps and institutional affiliations.

## Supplementary Material

Supplementary Information Supplementary Figures 1-8, Supplementary Table 1, Supplementary Notes 1-8 and Supplementary References

Peer Review File
