# Supplementary material for: Phosphorene/rhenium disulfide heterojunction-based negative differential resistance device for multi-valued logic
Source: Nat Commun. 2016 Nov 7;7:13413. doi: 10.1038/ncomms13413 (PMC5103069; doi:10.1038/ncomms13413)
Supplement: Supplementary Information — Supplementary Figures 1-8, Supplementary Table 1, Supplementary Notes 1-8 and Supplementary References [file ncomms13413-s1.pdf]

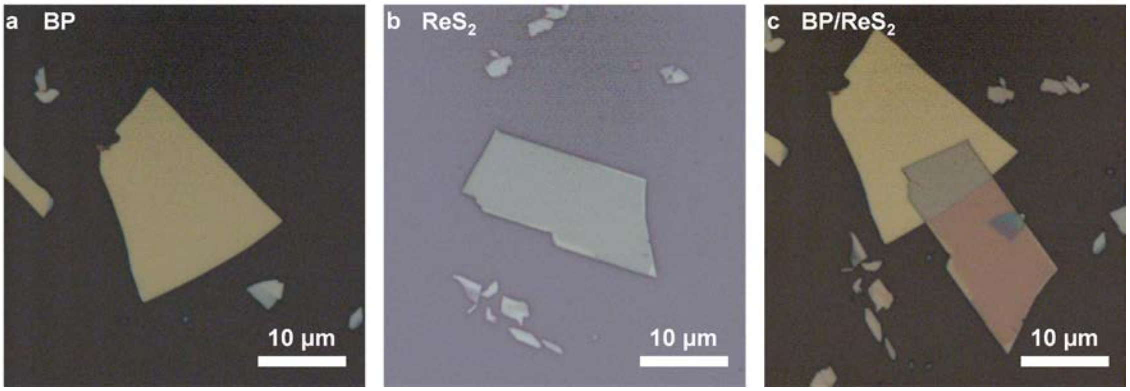


**Supplementary Figure 1. (a),(b),(c)** Optical images of exfoliated (a) BP and (b) $ReS_2$ flakes. (c) Optical image of $BP/ReS_2$ heterostructure fabricated by mechanically transferring the $ReS_2$ flake shown in (b) onto the BP flake shown in (a).

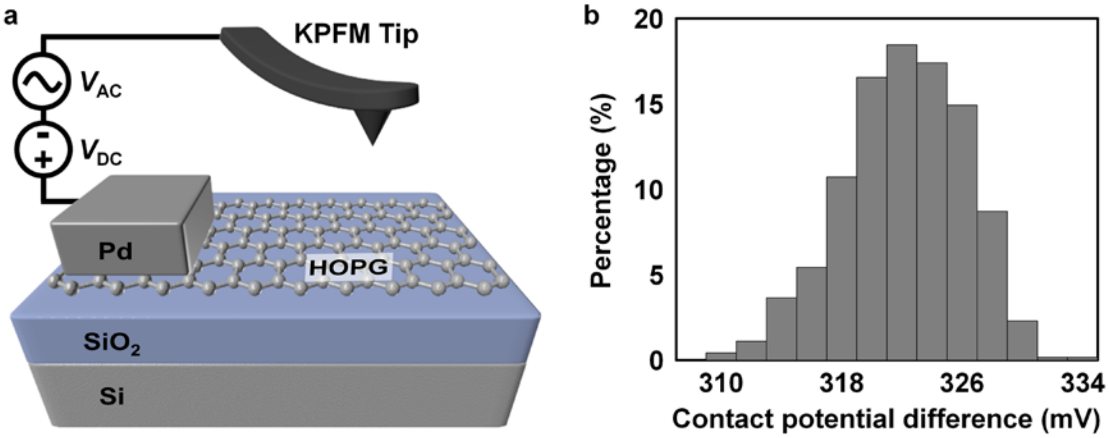


**Supplementary Figure 2. (a)** Schematic illustration of KPFM measurements. **(b)** Histogram of contact potential difference of the HOPG surface.

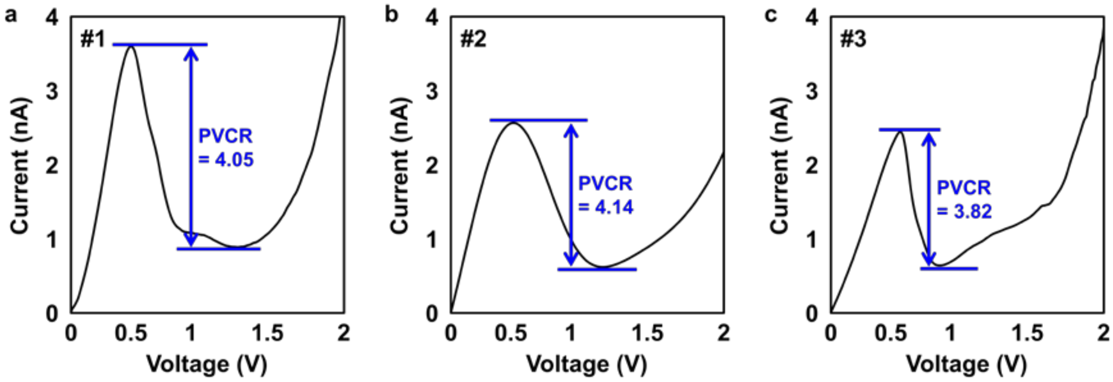


**Supplementary Figure 3. (a)–(c)** Current–voltage characteristics of the BP/$ReS_2$ NDR devices.

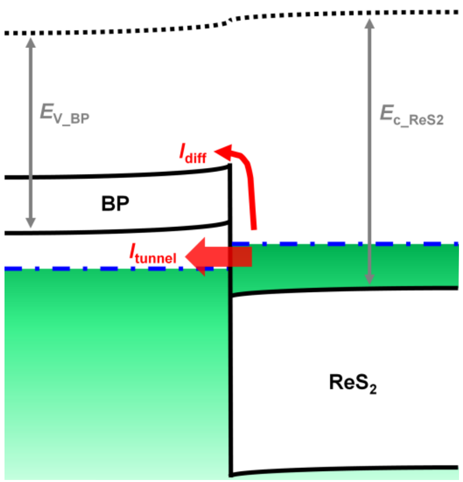


**Supplementary Figure 4.** Energy band diagram of the BP/$ReS_2$ heterojunction under a positive $V_{DS}$.

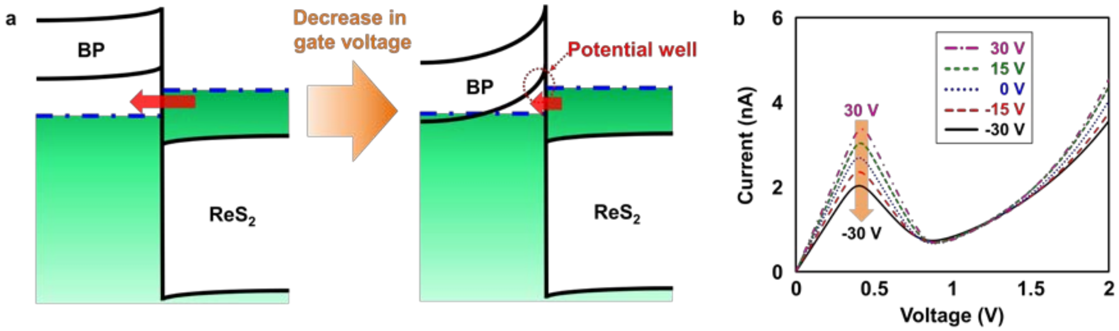


**Supplementary Figure 5. (a)** Energy band diagrams of the BP/$ReS_2$ heterojunction with decreasing gate voltage. **(b)** Theoretically calculated $I_D$-$V_D$ curves of the BP/$ReS_2$ NDR device as gate voltage decreases from 30 V to –30 V.

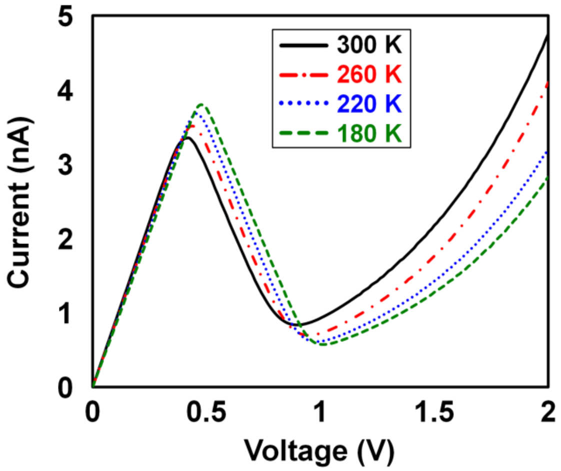


**Supplementary Figure 6.** Theoretically calculated *I*-*V* characteristic curves of the BP/$ReS_2$ NDR device at temperatures of 300 K, 260 K, 220 K, and 180 K.

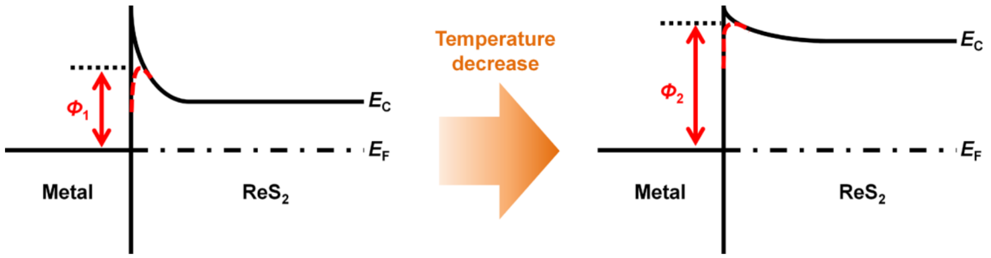


**Supplementary Figure 7.** Energy band diagrams of metal/$ReS_2$ junctions at high temperature (left) and low temperature (right). $E_C$ and $E_F$ are the lowest energy levels of the conduction band and the Fermi level in $ReS_2$, respectively. $\Phi_1$ and $\Phi_2$ indicate the effective barrier heights between metal and $ReS_2$.

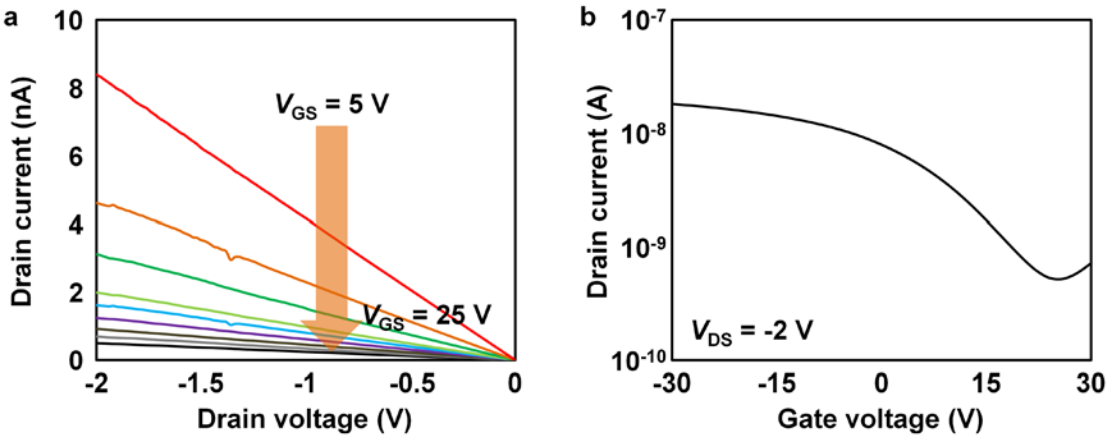


**Supplementary Figure 8.** (a) $I_D$-$V_D$ curves of the BP TFT under various gate voltages (5 V to 25 V). (b) $I_D$-$V_G$ curve of the BP TFT at $V_{DS}$ = -2 V.

**Supplementary Table 1.** The parameters used for the analytic NDR device model.

| $E_{V\_BP}$ [eV] | $E_{C\_Re}$ [eV] | $E_{F\_BP}$ [eV] | $E_{F\_Re}$ [eV] | $E_{g\_BP}$ [eV] | $E_{g\_Re}$ [eV] |
|---|---|---|---|---|---|
| 4.59* | 4.68* | 4.5 | 5.1 | 0.39* | 1.37* |
| **$m^*_{\_BP}$ [kg]** | **$m^*_{\_Re}$ [kg]** | **$\eta_{id}$** | **$\alpha$** | **$I_0$ [A]** | |
| $7.56\times10^{-31}$* | $2.71\times10^{-31}$* | 12 @ RT | 1.27 | $2.28\times10^{-10}$ @ RT | |

* The material parameters were obtained from those previously reported in the literature[4-8].

## Supplementary Note 1. Optical images of BP, $ReS_2$, and BP/$ReS_2$ heterostructurally stacked flakes

Supplementary Figures 1a–c show optical images of exfoliated black phosphorus (BP) on an $SiO_2$/Si substrate, rhenium disulfide ($ReS_2$) on a poly(methyl methacrylate) (PMMA)/polyvinyl alcohol (PVA)/Si substrate, and a BP/$ReS_2$ heterostructure on an $SiO_2$/Si substrate, respectively. First, the BP and $ReS_2$ flakes were exfoliated on $SiO_2$/Si and PMMA/PVA substrates, respectively. Then, the PVA layer was dissolved in water, and PMMA supported-$ReS_2$ was mechanically transferred onto the BP flake, followed by removal of the PMMA layer in acetone.

## Supplementary Note 2. Work function calibration of KPFM tip

A schematic illustration of the Kelvin probe force microscopy (KPFM) measurements is shown in Supplementary Figure 2a. To calibrate the work function of the KPFM tip, the KPFM measurement was performed on a highly oriented pyrolytic graphite (HOPG) surface. The obtained contact potential difference ($\Delta V_{CPD}$), which is the difference in work function between the KPFM tip and the sample, was between 310 mV and 334 mV. Because the work function of HOPG ($\Phi_{HOPG}$) is known to be 4.6 eV[1], the KPFM tip work function ($\Phi_{tip}$) can be obtained as the sum of the $\Phi_{HOPG}$ and the $\Delta V_{CPD}$ ($\Phi_{tip}$= 4.6 + $\Delta V_{CPD}$). As a result, we found that the work function of the KPFM tip was about 4.92 eV.

## Supplementary Note 3. I-V characteristics of the BP/$ReS_2$ NDR devices

We carried out current–voltage ($I$-$V$) measurements on three different BP/$ReS_2$ NDR devices. As shown in Supplementary Figure 3, similar electrical characteristics were observed in all of the devices, where the peak-to-valley current ratio (PVCR) values were between 3.82 and 4.14.

## Supplementary Note 4. Energy band diagram of the BP/$ReS_2$ heterojunction to explain the carrier transport mechanism

Supplementary Figure 4 shows an energy band diagram of the BP/$ReS_2$ heterojunction NDR device including important parameters that are required for the theoretical analysis of the electrical characteristics. Here, we considered tunneling current ($I_{tunnel}$) and diffusion current ($I_{diff}$) to understand the operation mechanisms of the BP/$ReS_2$ NDR device. $I_{tunnel}$ and $I_{diff}$ can be obtained from[2,3]

$$I_{\text{tunnel}} = \frac{2\pi\alpha q}{h}\int_{E_{\text{C\_Re}}}^{E_{\text{V\_BP}}} DOS_{\text{BP}}(E)\times DOS_{\text{Re}}(E)\times[f_{\text{BP}}(E) - f_{\text{Re}}(E - q(V - IR_{\text{s}}))]dE$$

(Supplementary Equation 1)

$$I_{\text{diff}} = qI_0[\exp(\frac{V - IR_{\text{s}}}{\eta_{id} k_{\text{B}} T}) - 1],$$ (Supplementary Equation 2)

where α is the screening factor, $q$ is the elementary charge, $h$ is Planck's constant, $E_{\text{V\_BP}}$ is the highest valence band energy in BP, $E_{\text{C\_Re}}$ is the lowest conduction band energy in $ReS_2$, $V$ is the applied voltage, $R_{\text{s}}$ is the series resistance, $I_0$ is the saturation current, $\eta_{\text{id}}$ is the ideality factor, $k_{\text{B}}$ is Boltzmann's constant, and $T$ is the temperature. $DOS_{\text{BP}}(E)$, $DOS_{\text{Re}}(E)$, $f_{\text{BP}}(E)$, and $f_{\text{Re}}(E)$ represent the density of states and Fermi-Dirac distribution functions of BP and $ReS_2$ respectively. Here, $DOS(E)$ is given by $(4\pi m^*)/h^2$, where $m^*$ is the effective mass of the 2D material. $f(E)$ is given by $(1+\exp[(E-E_{\text{F}})/k_B T])^{-1}$, where $E_{\text{F}}$ is the Fermi energy of the 2D material. The parameters used in the analytic model are tabulated in Supplementary Table 1.

**Supplementary Note 5. Energy band diagrams of the BP/$ReS_2$ heterojunction as gate voltage is reduced**

When the gate voltage decreases, the degree of the energy band bending in the BP region is increased due to the accumulated hole carriers. This subsequently causes a down-shift of the energy band in BP, as shown in Supplementary Figure 5a. This steep energy band bending in the BP region forms a potential well at the BP/$ReS_2$ heterojunction interface, thereby inducing a strong confinement of the electron carriers injected from the conduction band states in $ReS_2$. This eventually decreases the peak current of the BP/$ReS_2$ NDR device. As shown in Supplementary Figure 5b, the reduction of peak-current in BP/$ReS_2$ NDR devices with decreasing gate voltage could also be estimated using the $I_{\text{D}}$-$V_{\text{D}}$ curves calculated by the analytic model. In the analytic NDR device model, Fermi level modulation of the BP region *via* the gating effect was considered by assuming that the Fermi level shifts downward by 0.1 eV with decreasing gate voltage from 30V to –30 V (0.1 eV per 60 V = 0.00167 eV $V^{-1}$).

**Supplementary Note 6. Theoretically calculated electrical characteristics of the BP/$ReS_2$ NDR device at different temperatures**

Supplementary Figure 6 shows the theoretically calculated $I$-$V$ characteristic curves of the BP/$ReS_2$ NDR device at different temperatures (300 K, 260 K, 220 K, and 180 K) based on the

proposed analytic model shown in Supplementary Figure 4. Here, the total current of the BP/$ReS_2$ NDR device is expressed as the sum of the tunneling and diffusion currents. The calculated *I-V* curves were well fitted to the experimentally measured *I-V* curves (Figure 3a in main text).

**Supplementary Note 7. Energy band diagrams of the Metal/$ReS_2$ junctions as temperature decreases**

Supplementary Figure 7 shows the energy band diagrams of the metal/$ReS_2$ junctions at high temperature and low temperature. We consider the existence of parasitic series resistance ($R_S$) to accurately analyze the operation of the BP/$ReS_2$ NDR device. Specifically, the $R_S$ is primarily related to the contact resistance ($R_C$) between the metal electrode and the semiconductor, where $\Phi$ at the metal/$ReS_2$ junctions is an important parameter to determine the $R_C$ because electron carriers are injected from the source metal to $ReS_2$ when a positive voltage is applied on the drain metal electrode of the $ReS_2$ device. As temperature decreases, the n-type carrier concentration reduces and the Fermi level of $ReS_2$ becomes close to the intrinsic energy level, thereby increasing the depletion width at the metal/$ReS_2$ junction. This increased depletion width suppresses the barrier height lowering effect by reducing the electric field, consequently increasing the effective barrier height. Therefore, increasing voltage is required to operate the BP/$ReS_2$ NDR device with decreasing temperature because of the increased $R_C$ at the metal/$ReS_2$ junction.

**Supplementary Note 8. Electrical characteristics of the BP TFT**

Supplementary Figures 8a and b present the drain current–drain voltage ($I_D$-$V_D$) and drain current–gate voltage ($I_D$-$V_G$) characteristics of the BP TFT, respectively. These results show that p-channel behavior occurred in the BP TFT. In addition, we confirmed that the BP TFT can be used as a variable resistor that is controlled by an applied gate voltage. We obtained the on/off-current ratio as 400 A/A, and the threshold voltage of the BP TFT as 16 V.

## Supplementary References


1. Takahashi, T., Tokailin, H. & Sagawa, T. Angle-resolved ultraviolet photoelectron spectroscopy of the unoccupied band structure of graphite. *Phys. Rev. B* **32**, 8317-8324 (1985).

2. Roy, T. *et al.* Dual-Gated $MoS_2/WSe_2$ van der Waals Tunnel Diodes and Transistors. *ACS Nano* **9**, 2071-2079 (2015).

3. Smith, J. T., Das, S. & Appenzeller, J. Broken-Gap Tunnel MOSFET: A Constant-Slope Sub-60-mV/decade Transistor. *IEEE Electron Dev. Lett.* **32**, 1367-1369 (2011).

4. Perello, D. J., Chae, S. H., Song, S. & Lee, Y. H. High-performance n-type black phosphorus transistors with type control via thickness and contact-metal engineering. *Nat. Commun.* **6**, 7809 (2015).

5. Liu, X. *et al.* Black Phosphorus Based Field Effect Transistors with Simultaneously Achieved Near Ideal Subthreshold Swing and High Hole Mobility at Room Temperature. *Sci. Rep.* **6**, 24920 (2016).

6. Ho, C. H., Huang, Y. S., Chen, J. L., Dann, T. E. & Tiong, K. K. Electronic structure of $ReS_2$ and $ReSe_2$ from first-principles calculations, photoelectron spectroscopy, and electrolyte electroreflectance. *Phys. Rev. B* **60**, 15766-15771 (1999).

7. Liu, H. *et al.* Phosphorene: An Unexplored 2D Semiconductor with a High Hole Mobility. *ACS Nano* **8**, 4033-4041 (2014).

8. Yu, Z. G., Cai, Y. & Zhang, Y.-W. Robust Direct Bandgap Characteristics of One- and Two-Dimensional $ReS_2$. *Sci. Rep.* **5**, 13783 (2015).
